# Supplementary figures and images for: Tauroursodeoxycholic Acid Confers Protection Against Oxidative Stress via Autophagy Induction in Retinal Pigment Epithelial Cells
Source: Curr Issues Mol Biol. 2025 Mar 26;47(4):224. doi: 10.3390/cimb47040224 (PMC12025947; doi:10.3390/cimb47040224)

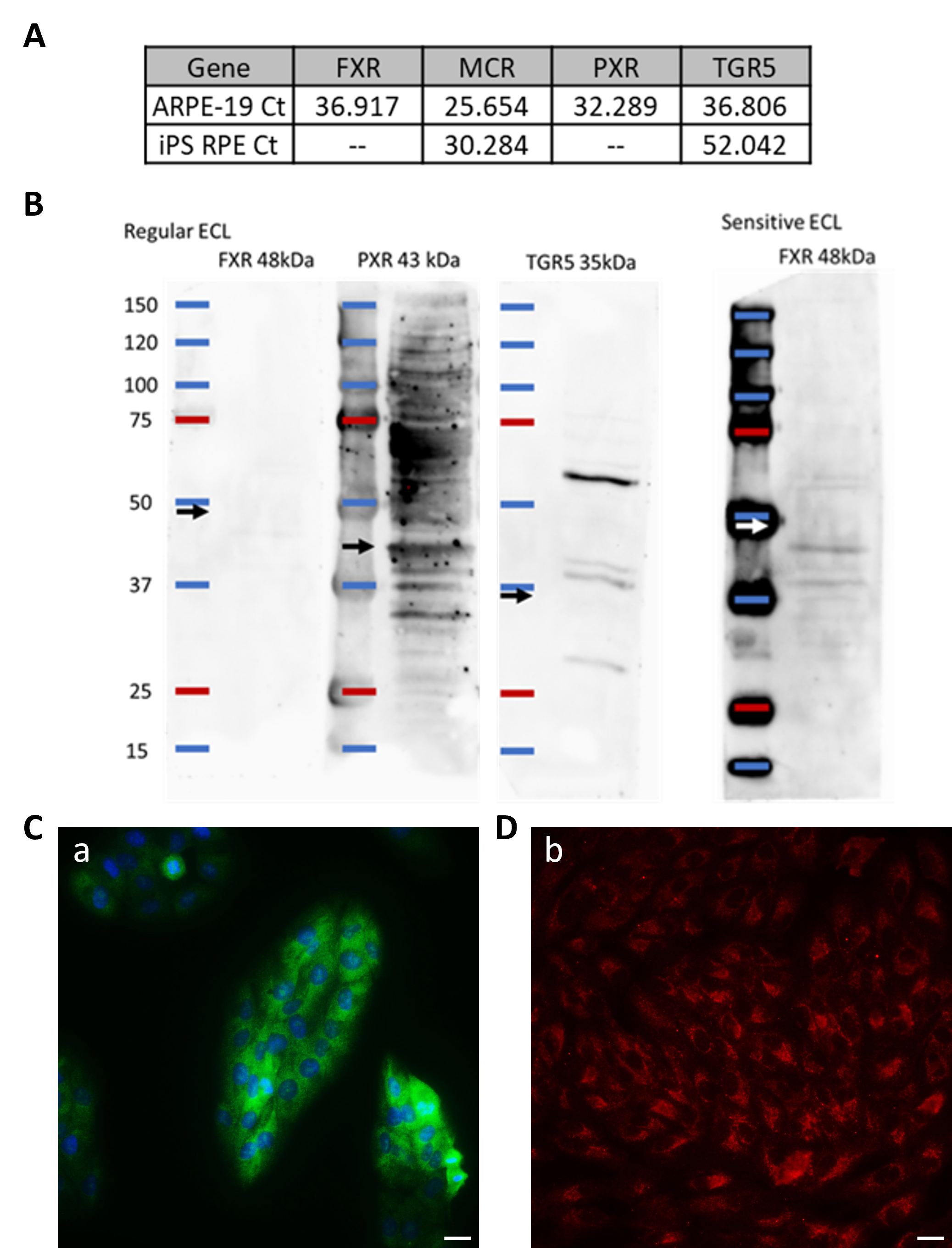

Supplement: Supplementary file 1 [file cimb-47-00224-s001.zip › Sup Fig 1.tif]

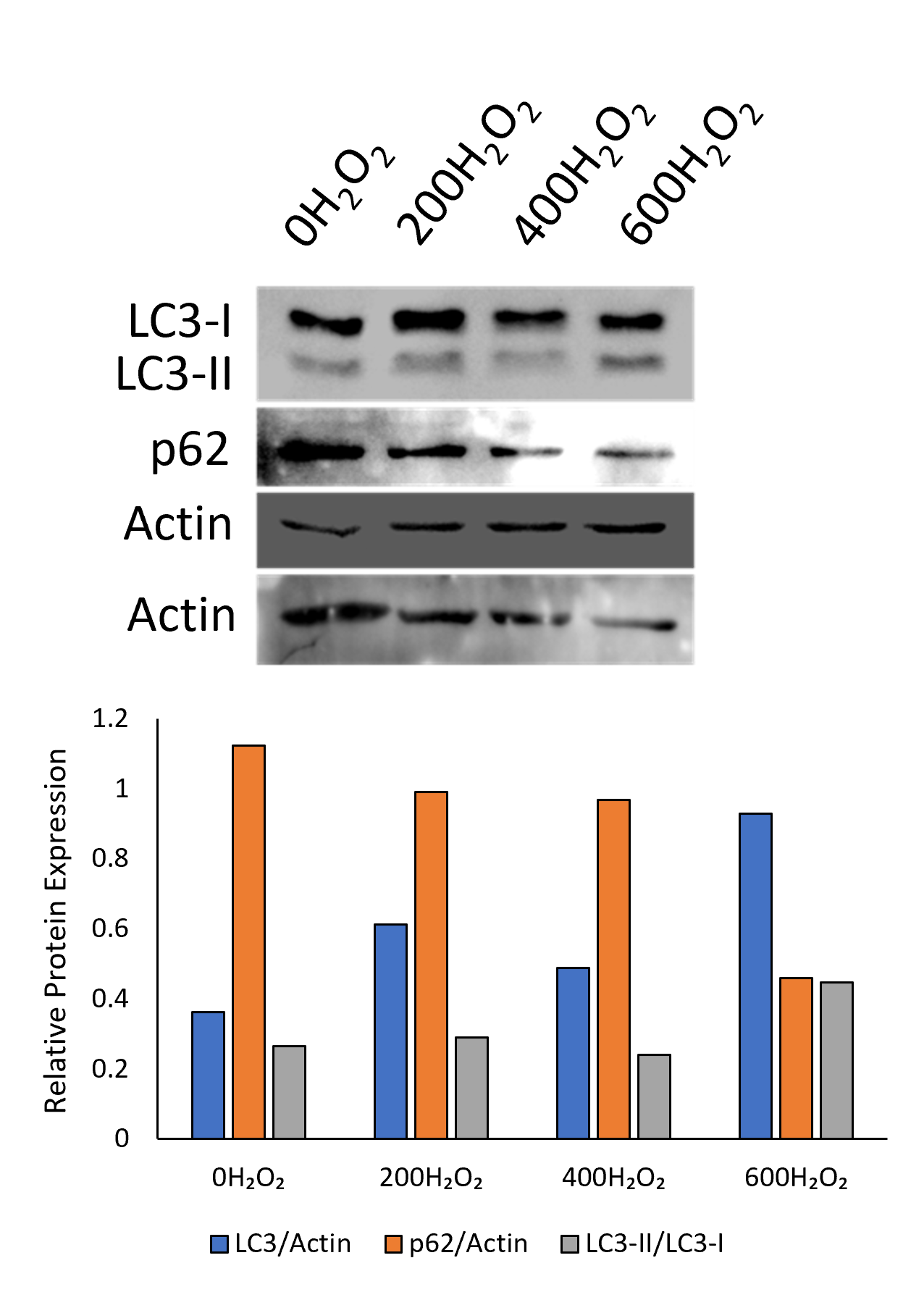

Supplement: Supplementary file 1 [file cimb-47-00224-s001.zip › Sup Fig 2.tif]
